# Decrease of 7T MR short-term effects with repeated exposure

**DOI:** 10.1007/s00234-024-03292-4

**Published:** 2024-01-25

**Authors:** Boel Hansson, Benjamín Garzón, Martin Lövdén, Isabella M Björkman-Burtscher

**Affiliations:** 1 Department of Medical Imaging and Physiology, Skåne University Hospital, Lund, Sweden; 2 Department of Diagnostic Radiology, Clinical Sciences, Lund University, Lund, Sweden; 3 Institute of Education, University of Zurich, Zurich, Switzerland; 4 Department of Psychology, Gothenburg University, Gothenburg, Sweden; 5 Aging Research Center, Department of Neurobiology, Care Sciences and Society, Karolinska Institute, Stockholm, Sweden; 6 Department of Radiology, Institute of Clinical Sciences, Sahlgrenska Academy, University of Gothenburg, Gothenburg, Sweden; 7 Department of Radiology, Sahlgrenska University Hospital, Region Västra Götaland, Gothenburg, Sweden

**Keywords:** Magnetic resonance imaging, Dizziness, Nausea, Adaptation, Biological

## Abstract

**Purpose:**

Although participants in 7 T magnetic resonance (MR) studies tolerate ultra-high field (UHF) well, subjectively experienced short-term effects, such as dizziness, inconsistent movement, nausea, or metallic taste, are reported. Evidence on subjectively experienced short-term effects in multiple exposures to UHF MR is scarce. The purpose of this study is to investigated experience of short-term effects, and occurrence of motion in healthy subjects exposed to seven weekly 7 T MR examinations.

**Methods:**

A questionnaire on short-term effects was completed by participants in an fMRI motor skill study. Seven UHF MR examinations were conducted over 7 weeks (exposure number: 1 to 7). Changes of experienced short-term effects were analyzed. Motion in fMRI images was quantified.

**Results:**

The questionnaire was completed 360 times by 67 participants after one to seven 7T MR examinations. Logistic mixed model analysis showed a significant association between dizziness, inconsistent movement, nausea, and headache and the examination numbers (*p*<0.03). Exposure to repeated examinations had no significant effect on peripheral nerve stimulation (PNS) or motion of the subjects. The overall experience of a 7T examination improved significantly (*p*<0.001) with increasing examination numbers.

**Conclusion:**

During multiple 7T examinations, subjects adapt to the strong static field. The short-term effects dizziness, inconsistent movement, nausea, and headache decrease over time as the MR sessions continue and experienced comfort increases. There was no significant difference in motion during the multiple fMRI examinations.

## Introduction

Although subjectively experienced short-term effects (e.g., dizziness, inconsistent movement, nausea, or metallic taste) are frequently reported by 7 Tesla (T) magnetic resonance (MR) study participants and patients, these populations have been shown to tolerate ultra-high field (UHF) strengths well [1–6]. Dizziness and inconsistent movement are suggested to result from a Lorentz force acting on the vestibular system. This may influence subjects’ perception of short-term effects during movement in and out of the static magnetic field in the scanner. Nausea is a consequence of dizziness [7–9]. A significant increase in occurrence of dizziness has been detected between 7 T and 1.5 T [3]. Metallic taste is most often described to originate from direct stimulation of the taste buds and electrolysis of saliva [1, 10]. Headache might be caused by several factors. Dizziness, noise and pressure to the head inside the coil have been sugested [8]. Further, psychological factors such as stress and anxiety related to the examination or its outcome are known factors to cause headache, influence compliance, or change the experience of short-term effects in general [6]. Peripheral nerve stimulation originates from the fast changing time varying gradient field and manifests as tingling or, in rare cases, painful muscle contractions [11]. Short-term effects, dizziness and nausea, similar to those experienced during UHF MR can also be provoked by other stimuli and adaptation based on repeated exposure has been reported using sessions of virtual reality (VR) simulation of a rollercoaster motion [12]. Habituation with visospatial training has also been shown to reducing motion sickness [13]. Physical and psychological comfort increase compliance with examinations and thus also reduce involuntary motion [14].

UHF MR currently plays a key role in neuroscience and preclinical research on disease pathology. Its significance in clinical diagnostics is expanding as more approved systems are installed and added clinical value is validated and established [6, 15, 16]. Multiple exposures to UHF strengths become thus more likely for larger patient populations. Although studies have shown that 7 T examinations are well tolerated in both patients and healthy research subjects [1–6], short-term effects still need to be considered when 7 T MR is used in clinical routine.

Evidence on subjectively experienced short-term effects in subjects with multiple exposure to UHF systems is scarce [4, 17], especially for repetitive exposure with rather short intervals. Knowledge of how multiple exposures are experienced is important for handling follow-up examinations in a clinical setting.

## Aim

The aim of this study was to investigate potential adaptation to repeated UHF exposure in healthy research subjects considering short-term effects and to evaluate attitude towards 7 T MR and occurrence of motion artefacts in a population of research subjects undergoing seven 7 T MR examinations in 7 weeks.

## Material and method

Participants from a 7 T fMRI motor skill randomized controlled trial (RCT) focusing on finger sequence execution coupled to training and including seven 7 T functional and structural MR examinations with 1-week intervals were also asked to participate in this study by completing a web-based questionnaire after each of the seven MR examinations performed in each subject. Repeated MR scans were run in five waves, each comprising 14 out of 70 healthy right-handed adults (20–31 years old) enrolled [17].

### Data collection

After each 7 T MR examination, a web-based questionnaire (REDCap; research electronic data capture; http://project-redcap.org) was used to collect data on subject demographics, on short-term effects experienced, on attitude towards the 7 T MR examination, on perception of the experience, and on perception of the MR examination in question in comparison to potential previous MR examinations. Adjectival and bipolar Likert scales [18] were used as detailed in the questionnaire given in Table 1. Experience of the short-term effects dizziness, inconsistent movement, nausea, headache, and metallic taste were evaluated concerning four situations: lying on the table and moving into the scanner (IN), being at the isocenter (INSIDE), moving out of the scanner (OUT), and being outside of the scanner after the examination (OUTSIDE), in accordance with a previous study [15]. Peripheral nerve stimulation (PNS) was evaluated regarding occurrence and perceived intensity and discomfort.

**Table 1:** Used questionnaire. In addition to examination date and study ID the follow questions relevant for this study were posed

Question	Used scale and scores
If you have had previous MR-examinations, how do you remember the last examination?	Five-step bipolar Likert scale (have not had any previous MR-examination, not uncomfortable at all, very little uncomfortable, little uncomfortable, moderate uncomfortable, very uncomfortable, very much uncomfortabel)
Did you experience *inconsistent movement* when *going INTO* the scanner? Question repeated for: *dizziness*, *nausea*, *headache*, and *metallic taste*, respectively; and questions were further repeated for: *INSIDE* the scanner, *OUT* of the scanner, and *OUTSIDE* the scanner, respectively.	Six-grade adjectival scale scale (none experienced, very little, little, moderate, much, very much)
Did you experience any twitches in any body part during the examination? If you felt twitches in any body part during the examination, how intensely did the twitches feel when most severe?	Six-grade adjectival scale (none experienced, very little, little, moderate, much, very much)
If you experienced any twitches, how would you rate this experience?	Six-grade adjectival scale scale (did not experience any twitches, not uncomfortable at all, very little uncomfortable, little uncomfortable, moderate uncomfortable, very uncomfortable, very much uncomfortable)
Would you rate the total experience of this MR-examination as comfortable?	Five-step bipolar Likert scale scale (strongly agree, agree, neither agree nor disagree, disagree, strongly disagree)
If you have had any previous MR-examinations, how would you rate todays’ MR-examination compared to your last one?	Three-step bipolar Likert scale scale (have not had any previous MR, better than previous, same as previous, worse than previous)
How would you describe your attitude towards today’s examination when you arrived at the department? How would you describe your attitude towards today’s examination now that the examination is completed?	Five-point adjectival scale scale (very much positive, much positive, moderate positive, little positive, very little positive, not positive at all)

Frame wise displacement (FD) and DVARS (DVARS, where D, temporal derivate of time courses, VARS, variance of root mean square (RMS)) [19, 20] were collected for the purpose of motion correction in the analysis of the fMRI data. These measures were used in the present study to analyze movement of participants and potential change of movement severity based on how many examinations the participant had undergone.

### MR system

Examinations included in the motor skill RCT were conducted in an actively shielded 7 T MR scanner (Achieva; Philips, Best, the Netherlands). The protocol included a structural T2-weighted sequence, an MP2RAGE sequence, and five runs of an 8-min fMRI sequence. This resulted in a total acquisition time of 55 min. In a previous study (*n* = 44 subjects) actual mean predicted PNS values was reported for the used scanner and the structural T2 and MP2RAGE sequences to correspond to 82%, and to 84% for the fMRI sequence, respectively [17]. For an 80-kg male and a 70 kg female average person the approximated predicted PNS values for the structural T2, the MP2RAGE and the fMRI sequence on the scanner are 67%, 51%, and 73% respectively.

### Statistics

Descriptive statistics: mean and range were used to present demographics. A logistic mixed model was used to analyze differences in experience of short-term effects between the repeated examinations, and odds ratio (OR) with 95% confidence interval (CI) was used to model the probability of experiencing short-term effects at each of the seven repeated 7 T MR examinations. For each short-term effect bar charts were used to show the distribution of the mean maximum values for the four locations (IN, INSIDE, OUT, and OUTSIDE). In addition, a linear mixed model analysis was used in comparison of the number of 7 T MR examinations and attitude prior to the MR examination, attitude after the MR examination, the comfort of the overall experience, and comparison of previous MR to this 7 T examination. A linear mixed model analysis for mean FD and DVARS in the five fMRI runs was used to analyze the effect of the number of repeated examinations on the involuntary motion of the subjects. Mann-Whitney *U*-test was used to evaluate potential drop out bias for participants not answering all 7 questionnaires. Any *p*-value ≤0.05 was regarded as being statistically significant. We used SPSS version 28 (IBM, Armonk, NY, US) and Stata Statistical Software: Release 16. (College Station, TX, US: StataCorp).

## Results

**Fig. 1:**
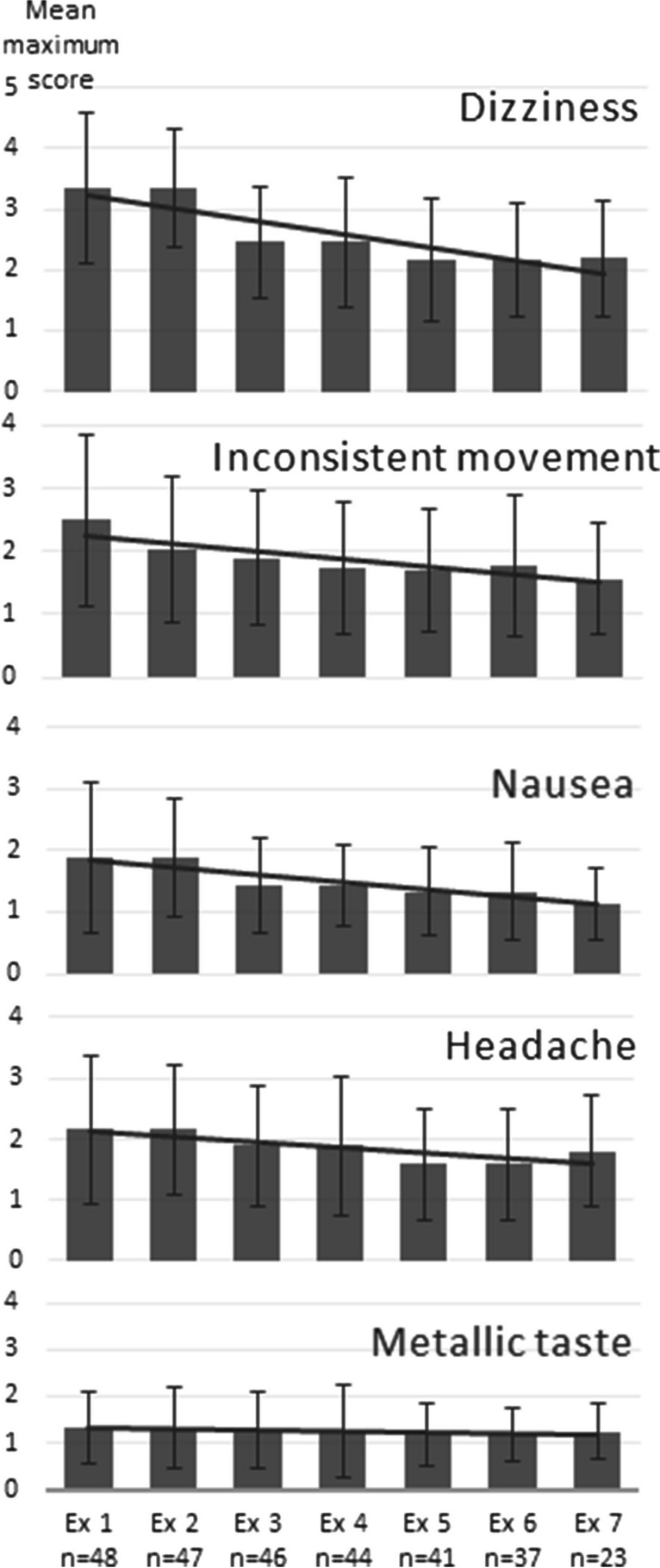
Short-term effects given as mean of maximum scores (0–5) for the four positions IN/INSIDE/OUT/OUTSIDE per individual and MR examination, standard deviation, and trend. Examination (Ex) 1–7 indicates the MR examination order, and n is the number of participants filling out the questionnaire for each examination. A trendline is added to show the general direction of the experienced short-term effect over time

**Fig. 2:**
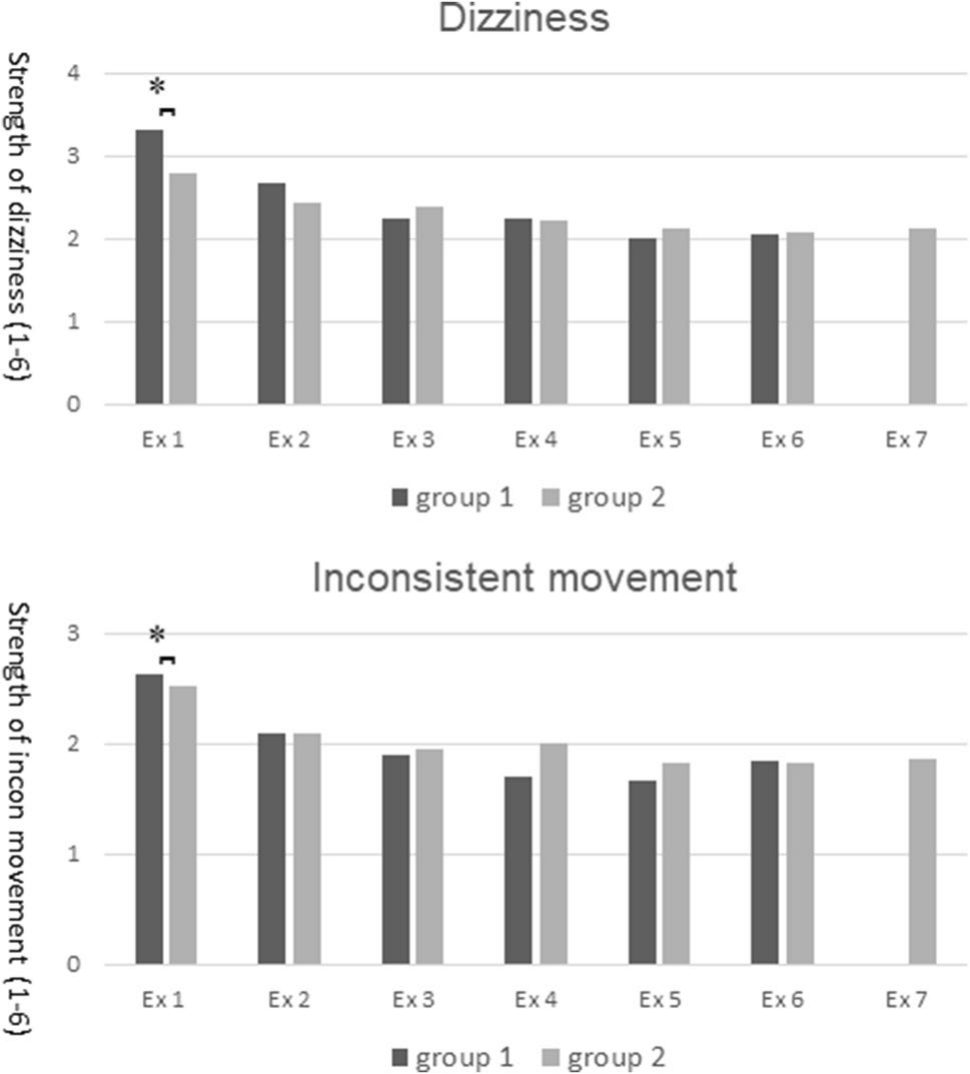
Drop out bias evaluation showing data for short-term effects dizziness (upper diagram) and inconsistent movement (lower diagram) for the 23 participants who answered all seven questionnaires (group 2) and for the rest of the participants answering the questionnaire after examination (Ex) 1 to 6, respectively, (group 1). * *p*=0.04. Despite the fact that group 2 experiences dizziness significantly less severely during examination 1 this trend does not persist compared to those participants dropping out from the study

Three subjects from the motor skill RCT did not consent to participate in this study. The remaining 67 participants (25 men and 41 women, mean age 25 years, range 20–30 years) rendered 360 completed questionnaires (in average 5 responses per participant) after one to seven 7 T MR examinations. Thirty-three of our participants were in the RCT randomized to the intervention group and thirty-three to the control group. In the last examination, 13 participants were from the intervention group and 10 from the control group. Thirty-eight participants had no previous MR examination. Dropout numbers for this study and the RCT are detailed in Table 2. Bar charts of the mean maximum values for IN, INSIDE, OUT, and OUTSIDE of the short-term effects dizziness, inconsistent movement, nausea, headache, and metallic taste as a function of MR examination number 1 to 7 are shown in Fig. 1 with a clear trend towards lower effects over time, except for metallic taste. The logistic mixed model analysis showed a significant association of MR examination number with dizziness IN, INSIDE, OUT, and OUTSIDE (*p*≤0.006), inconsistent movement IN and INSIDE, (*p*≤0.006), nausea IN, INSIDE, and OUTSIDE (*p*≤0.03), and headache IN, INSIDE, OUT, and OUTSIDE (*p*≤0.03). The analyses for dizziness, inconsistent movement, nausea, headache, and metallic taste are shown in detail in Table 3. The experience of short-term effects of the 23 participants who answered all seven questionnaires did not significantly differ from the experiences of the other participants at any given timepoint except for dizziness at the first examination (*p*=0.04) (Fig. 2).

**Table 2:** Distribution of undergone 7 T MR examinations and completed questionnaires for the 67 participants completing a total of 360 questionnaires

		Number of participants who underwent n (1–7) MR examinations								
		1	2	3	4	5	6	7	Participants (n)	Completed questionnaires (n)
Number of participants who completed n (1-7) questionnaires	1	1	0	0	0	0	3	1	5	5
	2	0	1	0	0	0	0	1	2	4
	3	0	0	1	0	0	1	2	4	12
	4	0	0	0	0	0	3	3	6	24
	5	0	0	0	0	1	3	4	8	40
	6	0	0	0	0	0	5	14	19	114
	7	0	0	0	0	0	0	23	23	161
Total		1	1	1	0	1	14	48	67	360

**Table 3:** Linear mixed model analysis for dizziness, inconsistent movement, nausea, headache, and metallic taste compared to the MR examination order; the table shows how much the response variables (dizziness, inconsistent movement, nausea, headache, and metallic taste) changes depending on the order of the examinations. *OR* odds ratio, *CI* confidence interval

	IN		INSIDE		OUT		OUTSIDE	
Order	OR (95 % CI)	*p*-value	OR (95 % CI)	*p*-value	OR (95 % CI)	*p*-value	OR (95 % CI)	*p*-value
Dizziness								
1	Reference		Reference		Reference		Reference	
2	0.948 (0.280 - 3.203)		0.696 (0.286 - 1.696)		0.731 (0.304 - 1.759)		0.395 (0.152 - 1.027)	
3	0.634 (0.192 - 2.091)		0.362 (0.145 - 0.905)		0.332 (0.134 - 0.822)		0.291 (0.109 - 0.776)	
4	0.169 (0.051 - 0.555)		0.169 (0.063 - 0.452)		0.437 (0.176 - 1.085)		0.087 (0.030 - 0.256)	
5	0.119 (0.035 - 0.403)		0.181 (0.066 - 0.496)		0.146 (0.052 - 0.405)		0.108 (0.036 - 0.324)	
6	0.113 (0.030 - 0.417)		0.070 (0.022 - 0.223)		0.229 (0.081 - 0.654)		0.118 (0.037 - 0.380)	
7	0.281 (0.061 - 1.305)		0.709 (0.197 - 2.554)		0.439 (0.124 - 1.553)		0.212 (0.054 - 0.833)	
		0.001		<0.001		0.006		<0.001
Inconsistent movement								
1	Reference		Reference		Reference		Reference	
2	0.249 (0.075 - 0.825)		0.613 (0.212 - 1.774)		0.845 (0.262 - 2.721)		0.168 (0.039 - 0.720)	
3	0.195 (0.058 - 0.662)		0.299 (0.098 - 0.915)		0.420 (0.212 - 2.441)		0.139 (0.031 - 0.632)	
4	0.102 (0.028 - 0.368)		0.150 (0.045 - 0.493)		0.720 (0.212 - 2.441)		0.076 (0.014 - 0.412)	
5	0.083 (0.022 - 0.324)		0.260 (0.079 - 0.858)		0.192 (0.049 - 0.749)		0.060 (0.010 - 0.374)	
6	0.078 (0.018 - 0.332)		0.078 (0.020 - 0.303)		0.296 (0.074 - 1.188)		0.066 (0.010 - 0.431)	
7	0.053 (0.008 - 0.364)		0.260 (0.053 - 1.276)		0.297 (0.047 - 1.878)		0.025 (0.002 - 0.402)	
		0.003		0.006		0.2		0.02
Nausea								
1	Reference		Reference		Reference		Reference	
2	0.375 (0.120 - 1.172)		0.584 (0.212 - 1.603)		0.584 (0.147 - 2.325)		0.287 (0.068 - 1.209)	
3	0.218 (0.063 - 0.753)		0.189 (0.057 - 0.627)		0.552 (0.134 - 2.266)		0.106 (0.020 - 0.566)	
4	0.160 (0.043 - 0.597)		0.268 (0.084 - 0.855)		0.569 (0.133 - 2.425)		0.103 (0.018 - 0.574)	
5	0.153 (0.038 - 0.616)		0.222 (0.063 - 0.787)		0.211 (0.037 - 1.210)		0.068 (0.010 - 0.470)	
6	0.097 (0.019 - 0.497)		0.135 (0.031 - 0.589)		0.061 (0.006 - 0.612)		0.017 (0.001 - 0.239)	
7	0.139 (0.019 - 1.001)		0.093 (0.012 - 0.713)		0.140 (0.012 - 1.643)		0.043 (0.003 - 0.636)	
		0.03		0.02		0.3		0.02
Headache								
1	Reference		Reference		Reference		Reference	
2	2.950 (1.067 - 8.159)		1.598 (0.683 - 3.739)		2.449 (0.933 - 6.428)		0.802 (0.334 - 1.929)	
3	0.567 (0.177 - 1.820)		0.808 (0.341 - 1.915)		0.999 (0.366 - 2.725)		0.663 (0.269 - 1.631)	
4	0.434 (0.125 - 1.507)		0.345 (0.137 - 0.871)		0.675 (0.235 - 1.938)		0.285 (0.106 - 0.767)	
5	0.185 (0.041 - 0.831)		0.204 (0.074 - 0.560)		0.190 (0.052 - 0.689)		0.206 (0.071 - 0.601)	
6	0.462 (0.123 - 1.736)		0.300 (0.108 - 0.830)		0.883 (0.287 - 2.718)		0.289 (0.099 - 0.844)	
7	0.419 (0.079 - 2.212)		0.449 (0.126 - 1.601)		0.347 (0.074 - 1.622)		0.355 (0.092 - 1.362)	
		0.003		0.001		0.009		0.03
Metallic taste								
1	Reference		Reference		Reference		Reference	
2	0.012 (0.000 - 1.719)		1.342 (0.225 - 7.985)		2.167 (0.193 - 24.548)		1.618 (0.255 - 10.252)	
3	0.108 (0.004 - 2.890)		0.640 (0.086 - 4.750)		0.398 (0.025 - 6.241)		0.136 (0.010 - 1.841)	
4	0.395 (0.024 - 6.533)		0.255 (0.024 - 2.710)		0.322 (0.017 - 6.078)		0.308 (0.029 - 3.301)	
5	0.135 (0.004 - 4.227)		1.250 (0.171 - 9.154)		1.272 (0.086 - 18.723)		1.536 (0.202 - 11.710)	
6	0.006 (0.000 - 2.988)		0.708 (0.081 - 6.195)		0.396 (0.019 - 8.280)		0.400 (0.034 - 4.741)	
7	1.026 (0.000 - 2.988)		0.821 (0.067 - 10.121)		1.507 (0.061 - 37.024)		0.955 (0.061 - 14.936)	
		0.6		0.9		0.9		0.5

Mean scores for quantity, intensity, and experience of PNS were low and did not differ significantly between examinations one to seven (*p*=0.9, p=0.3, and *p*=0.3, respectively) (Table 4). Results regarding experience of attitude prior to and after the MR examination, the overall comfort, and experience compared to previous MR are given in Table 5. Linear mixed model analysis showed a significant association between examination number and overall experience of comfort (*p*≤0.001), but not with attitude prior to the MR examination, and comparison to previous MR examinations (*p*=0.5, *p*=0.7) (Table 5). The overall experience of comfort in the 7 T MR examination was scored significantly (*p*<0.001) higher as the examination order increased. The head motion outcomes showed low levels of involuntary motion and no significant effect of the number of examinations on involuntary motion detected in fMRI scans. The linear mixed model analyses for mean FD and DVARS in all fMRI runs are shown in Table 6.

**Table 4:** Linear mixed model analysis for peripheral nerve stimulation (PNS) outcomes; the table shows how much the response variables (PNS quantity, intensity, and experience) change depending on the order of the examinations. Examination number 1 to 7 = order, *OR* odds ratio, *CI* confidence interval, *β* average difference

	PNS Quantity		PNS intensity		Experience of PNS	
Order	OR (95 % CI)	*p*-value	β (95 % CI)	*p*-value	β (95 % CI)	*p*-value
1	Reference		Reference		Reference	
2	1.173 (0.511 - 2.694)		-0.065 (-0.360 - 0.230)		0.041 (-0.257 - 0.339)	
3	1.229 (0.528 - 2.857)		-0.010 (-0.309 - 0.290)		-0.024 (-0.326 - 0.279)	
4	0.957 (0.406 - 2.259)		-0.084 (-0.390 - 0.222)		0.075 (-0.234 - 0.384)	
5	1.070 (0.441 - 2.597)		-0.033 (-0.348 - 0.282)		-0.011 (-0.330 - 0.307)	
6	0.646 (0.252 - 1.655)		-0.394 (-0.727 - -0.060)		-0.362 (-0.699 - -0.025)	
7	0.774 (0.244 - 2.454)		-0.203 (-0.618 - 0.211)		-0.112 (-0.530 - 0.307)	
		0.9		0.3		0.3

**Table 5:** Linear mixed model analysis for experience outcomes; the table shows how much the response variable changes (total experience, anxiety/stress, previous MR, and attitude prior to MR examination) depending on the order of the examinations. *CI* confidence interval, *β* average difference

	Total experience		Anxiety stress		Previous MR		Attitude prior to MR examination	
Order	β (95 % CI)	*p*-value	β (95 % CI)	*p*-value	β (95 % CI)	*p*-value	β (95 % CI)	*p*-value
1	Reference		Reference		Reference		Reference	
2	-0.154 (-0.351 - 0.043)		-0.289 (-0.497 - -0.082)		0.035 (-0.195 - 0.266)		0.049 (-0.166 - 0.265)	
3	-0.277 (-0.477 - -0.077)		-0.260 (-0.471 - -0.049)		0.087 (-0.148 - 0.323)		-0.013 (-0.237 - 0.212)	
4	-0.479 (-0.684 - -0.275)		-0.422 (-0.638 - -0.207)		0.058 (-0.181 - 0.298)		-0.026 (-0.255 - 0.204)	
5	-0.381 (-0.591 - -0.170)		-0.430 (-0.652 - -0.207)		0.185 (-0.064 - 0.433)		0.127 (-0.110 - 0.365)	
6	-0.402 (-0.625 - -0.179)		-0.388 (-0.624 - -0.153)		0.071 (-0.190 - 0.333)		-0.118 (-0.374 - 0.139)	
7	-0.338 (-0.616 - -0.060)		-0.457 (-0.750 - -0.164)		-0.195 (-0.537 - 0.147)		0.037 (-0.300 - 0.373)	
		<0.001		0.001		0.5		0.7

**Table 6:** Linear mixed model analysis for image correction outcomes; the table shows how much the response variables (mean FD, and mean DVARS) change depending on the order of the examinations. *CI* confidence interval, *β* average difference

	Mean FD		Mean DVARS	
Order	β (95 % CI)	*p*-value	β (95 % CI)	*p*-value
1	Reference		Reference	
2	-0.009 (-0.027 - 0.009)		0.902 (-0.990 - 2.794)	
3	-0.016 (-0.034 - 0.002)		0.333 (-1.584 - 2.250)	
4	-0.018 (-0.036 - 0.000)		0.248 (-1.711 - 2.208)	
5	-0.018 (-0.037 - 0.001)		0.285 (-1.748 - 2.318)	
6	-0.021 (-0.042 - -0.001)		0.645 (-1.508 - 2.799)	
7	-0.015 (-0.040 - 0.010)		1.134 (-1.545 - 3.813)	
		0.3		1.0

## Discussion

The experience of some short-term effects related to UHF MR changes in individuals with repeated examinations and some adaptation to the strong static magnetic field and the related MR environment occurs over time. This is also reflected in an increased perceived comfort over time. Further, these parameters did not impact on the ability to lie still in the MR scanner during the fMRI experiment.

Especially dizziness is a common short-term effect when moving through ultra-high MR fields with 60 to 80% of subjects reporting dizziness at 7 T [3, 20]. However, in studies with subjects attending multiple examinations a potential adaptation effect has not been considered [4, 17]. Our findings are of interest from a clinical perspective, as they indicate potential adaptation regarding some of the short-term effect when multiple 7 T sessions are performed. This is of great importance to demonstrate as 7 T now is a clinical routine application and will potentially be used also for clinical long-term follow-up of disease with multiple repeated examinations [15].

Our findings of adaptation of short-term effects are in line with adaptation findings in studies on motion sickness. Smyth et al. focused on the link between visuospatial skills and motion sickness and demonstrated that training visuospatial skills is an effective method of motion sickness management, and they are also highlighting that their findings might help in the management of “virtual reality sickness,” space sickness, sea sickness and other motion sickness states [13]. Adaptation effects have been demonstrated after 4–5 repeated sessions of VR training or short-term visuospatial training, and VR technology is suggested to be an effective tool for rehabilitation of visual vertigo [12]. Potential adaptation in our study was largest already after 2–3 repeated sessions. This study is not designed to explore potential physiological mechanisms leading to adaptation effects and this may be of interest in further studies.

Gratton et al. (2020) addressed the importance of removal of motion biases in fMRI and demonstrated that higher-frequency fluctuations in the motion parameters are more common in older adults, subjects with higher body mass index, and those with lower cardiorespiratory fitness. They suggest a framewise displacement (FD) approach to remove motion contamination [21]. We compared the magnitude of the motion measures (FD/DVARS) for the repeated MR examinations and found no significant difference in contamination both in FD and DVARS between the repeated MR examinations. Thus, 7T MR examinations and related short-term effects do not necessarily require test runs to prevent motion contamination; adaptation to UHF exposure over time may not necessarily affect motion contamination. However, it may be possible to minimize motion by making the conditions inside the scanner more comfortable for the research subject. Scan duration, auditory noise, and comfort are highlighted as issues [17]. Any MRI system may consider these points when setting up studies and clinical protocols.

As PNS is mainly related to the time varying magnetic field and an individual’s physical constitution [22], muscle twitches or pain related to PNS are not expected to adapt, as further confirmed in this study.

### Limitations

The number and intervals between measurement time points in this study were not optimized for the questions addressed in the present study but were dictated by the original RCT. For clinical populations, intervals of two to three months between examinations would have been more realistic. Additional aspects that distinguish this population from patients are for example age, medical history but also motivation to undergo an examination. Motivation is unpredictable and will differ within both research and patient populations based on for example anxiety related to diagnostic results, eagerness for a diagnostic breakthrough for an epilepsy patient, health condition and for example related pain or discomfort, reimbursement or lack of it for research studies, and a variety of psychological factors related to everyday problems and personal life situations. However, an advantage of using the chosen population was that participants did not primarily focus on the MR examination experience, but on the task at hand in the original RCT. Recognition bias and fatigue related to repetition might have influenced the quality and level of nuance in the responses. Similar to that, motivation of participants to answer all questionnaires differed between individuals and only 23 subjects participating in 7 MR examinations answered the questionnaire each time. Further, there was a similar dropout in the RCT, suggesting motivation fatigue regarding participation in the research studies. Drop out bias might also influence how the study population experiences short-term effects evaluated. However, the initial significant difference in experience of dizziness between those participating in all 7 examinations and questionnaires (less severe dizziness) does not persist at the other examinations and is not seen for any other short-term effect. We thus hypothesize that drop out from the study is not primarily mitigated by more severe short-term effects.

## Conclusion

Multiple UHF MR examinations are tolerated well and may lead to adaptation to the strong static field regarding short-term effects such as dizziness, inconsistent movement, and nausea. Experienced comfort increases over time. However, these effects do not have a significant impact on the ability to lie still and experience of peripheral nerve stimulation does not change.
